# Human precision-cut cystic duct and gallbladder slices: a novel method for studying cholangiopathies

**DOI:** 10.3389/fped.2023.1058319

**Published:** 2023-07-17

**Authors:** Jeske Fridrichs, Bart Hamel, Wendy Kelder, Ewoud van den Hoed, Marius C. van den Heuvel, Jan B. F. Hulscher, Peter Olinga

**Affiliations:** ^1^ Department of Pharmaceutical Technology and Biopharmacy, Groningen Research Institute of Pharmacy, University of Groningen, Groningen, Netherlands; ^2^ Division of Pediatric Surgery, Department of Surgery, University Medical Center Groningen, Groningen, Netherlands; ^3^ Department of Pathology, Martini Hospital, Groningen, Netherlands; ^4^ Department of Surgery, Martini Hospital, Groningen, Netherlands; ^5^ Department of Pathology, University Medical Center Groningen, Groningen, Netherlands

**Keywords:** precision-cut tissue slices (PCTS), cholangiopathies, *ex vivo* culture technique, cystic duct, gallbladder

## Abstract

**Background and aims:**

Precision-cut tissue slices (PCTS) are widely used as an *ex vivo* culture tissue culture technique to study pathogeneses of diseases and drug activities in organs *in vitro*. A novel application of the PCTS model may be in the field of translational research into cholangiopathies such as biliary atresia. Therefore, the aim of this study was to apply the precision-cut slice technique to human bile duct and gallbladder tissue.

**Methods:**

Cystic duct and gallbladder tissue derived from patients undergoing a cholecystectomy were collected, preserved and used for preparation of precision-cut cystic duct slices (PCCDS) and precision-cut gallbladder slices (PCGS). The PCCDS and PCGS were prepared using a mechanical tissue slicer and subsequently incubated for 24 and 48 h respectively in William's Medium E (WME) culture medium. Viability was assessed based on ATP/protein content and tissue morphology [hematoxylin and eosin (H&E) staining].

**Results:**

It was shown that viability, assessed by the ATP/protein content and morphology, of the PCCDS (*n* = 8) and PCGS (*n* = 8) could be maintained over the 24 and 48 h incubation period respectively. ATP/protein content of the PCCDS increased significantly from 0.58 ± 0.13 pmol/µg at 0 h to 2.4 ± 0.29 pmol/µg after 24 h incubation (*P *= .0003). A similar significant increase from 0.94 ± 0.22 pmol/µg at 0 h to 3.7 ± 0.41 pmol/µg after 24 h (*P* = .0005) and 4.2 ± 0.47 pmol/µg after 48 h (*P *= .0002) was observed in the PCGS. Morphological assessment of the PCCDS and PCGS showed viable tissue at 0 h and after 24 and 48 h incubation respectively.

**Conclusion:**

This study is the first to report on the use of the PCTS model for human gallbladder and cystic duct tissue. PCCDS and PCGS remain viable for an incubation period of at least 24 h, which makes them suitable for research purposes in the field of cholangiopathies, including biliary atresia.

## 1. Introduction

Precision-cut tissue slices (PCTS) are widely used as an *ex vivo* tissue culture technique to study pathogenesis and drug activities in organs *in vitro*. PCTS are very thin sections of an organ, which can be prepared using a mechanical tissue slicer. In PCTS, the different cell types present in the organ or tissue are left intact and the natural environment is preserved, which means natural intercellular and cell-matrix interaction are maintained (1). Therefore, PCTS can be regarded as the mini organ model that best resembles the *in vivo* situation. Tissue slicers are able to generate PCTS of a well-defined thickness, which ensures reproducibility of the PCTS (2). Viability of PCTS can be maintained for an incubation period of up to 120 h, depending on the species and tissue type (1). For example, human kidney slices and human lung slices remained viable up to 72 h, whilst human liver slices showed viability up to 120 h (3–5).

PCTS have been primarily used to study pharmacokinetic characteristics (absorption, distribution, metabolism, excretion and toxicity (ADME-tox) of drugs and xenobiotics. In addition to such ADME-tox studies, drug activities can be assessed with the use of tissue slices. Furthermore, PCTS have also been used for physiology and pathology-related research, for example on fibrosis and inflammatory processes, cancer and viral infections (6). Until now, the organs that have been used for preparation of PCTS are the liver, kidney, intestine, lungs, heart, spleen, brain, prostate and several types of tumor tissue, as extensively reviewed by de Graaf et al. (1).

In addition to its current applications, the PCTS model may also be a valuable model to study cholangiopathies such as biliary atresia (BA). BA is a rare obstructive cholangiopathy affecting the extrahepatic bile ducts of neonates. The etiology and pathogenesis of isolated BA is still poorly understood, but it appears to be a multifactorial interplay of genetic susceptibility, viral infections, environmental toxins and immune dysfunction (7). In support of an inflammatory component in its pathogenesis is the presence of an early circulating inflammatory process and T helper 17 cell infiltrates in the liver of children with BA (8, 9). Since the currently used animal and cell culture models have limitations that might impair translatability of findings to human cholangiopathies, a novel type of translational model is needed to gain further insights into the pathogenesis. Human PCTS may serve this purpose, as PCTS closely represent *in vivo* physiological conditions and can therefore be used for research into the pathogenesis of cholangiopathies. For example, in case of BA, PCTS may be used to study the effects of a viral trigger or toxin on the human bile duct and gallbladder.

Recently, human precision-cut bile duct slices (PCBDS) were prepared from the common bile duct and left and right hepatic ducts and used to study peribiliary glands and biliary regeneration in these ducts (10). These extrahepatic bile ducts can only be obtained from discarded donor livers or in rare major surgeries; hence availability of this tissue is limited. In this follow-up study, we used cystic duct samples from living subjects obtained during cholecystectomy procedures. Cholecystectomies, including the removal of a major part of the cystic duct, are performed as routine surgery. Therefore, cystic duct tissue can be relatively easily acquired. Another benefit of cholecystectomies as source of tissue is that patients undergoing this surgery are often relatively healthy and so is the removed tissue. The aim of this study was to develop the precision-cut slice technique for human cystic duct (PCCDS) and gallbladder (PCGS) tissue derived from cholecystectomies.

## 2. Methods

### 2.1. Tissue collection procedure

Gallbladder and cystic duct tissue derived from patients undergoing elective laparoscopic cholecystectomy for uncomplicated gallstone disease at the Martini Hospital (Groningen, The Netherlands) was used for preparation of the PCTS. To avoid interference with routine pathological histological assessment of the removed tissue, we used the tissue approximately 24 h after the cholecystectomy. Tissue was stored freshly after resection and send to the pathology department as soon as possible. There, the cystic duct remnant and part of the gallbladder were stored separately in University of Wisconsin organ preservation solution (UW solution) (11) in the refrigerator (4°C) until the next morning when the pathologist routinely examined the tissue for any signs of malignancy. If the tissue was declared free of abnormalities by the pathologist, the tissue could be used for the present study. Tissue was kept on ice at approximately 4°C during transport. Upon arrival at our lab, the tissue was immediately transferred into jars with clean UW solution (at 4°C) and stored on ice until processing.

The medical ethical committee of the Martini Hospital approved the anonymous use of gallbladder and cystic duct tissue (research registration number: 2019-114).

### 2.2. Preparation of the PCCDS and PCGS

Firstly, tissue cores had to be prepared. During preparation of the tissue cores, the tissue was kept wet with UW solution (at 4°C) to prevent degradation.

#### 2.2.1. Cystic duct tissue cores

**Figure 1:**
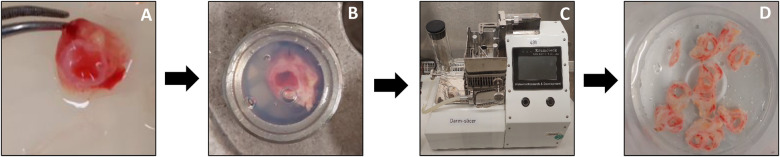
Preparation and slicing of human PCCDS. Remnant of cystic duct was flushed with UW solution, cleaned and as much of the loose connective tissue as possible was removed (**A**). The segment was placed in the core holder, which was filled with 3% low-gelling-temperature agarose (**B**). After solidification of the agarose, the core was removed from the core holder and placed in the Krumdieck tissue slicer (**C**). PCCDS were collected in UW solution during the slicing process (**D**) and afterwards transferred to culture plates. Tissue was kept at 4 degrees celsius during the whole process.

The first step in preparation of the tissue cores was removal of as much of the fat and loose connective tissue surrounding the cystic duct as possible. Subsequently, the cystic duct was flushed with UW solution in order to remove any remaining bile left in the duct. Next, the cystic duct segment was placed into the core holder, which was then filled quickly with 3% low-gelling-temperature agarose type VII (Sigma-Aldrich, Zwijndrecht, The Netherlands) at a temperature of approximately 37°C. Thereafter, the agarose was solidified by placing the core-holder on ice for at least 15 min. Afterwards, the core was ready to be used for slicing and was placed in the Krumdieck slicer (Alabama Research and Development, Munford, USA). Figure 1 provides an overview of the entire procedure.

#### 2.2.2. Gallbladder tissue cores

**Figure 2:**
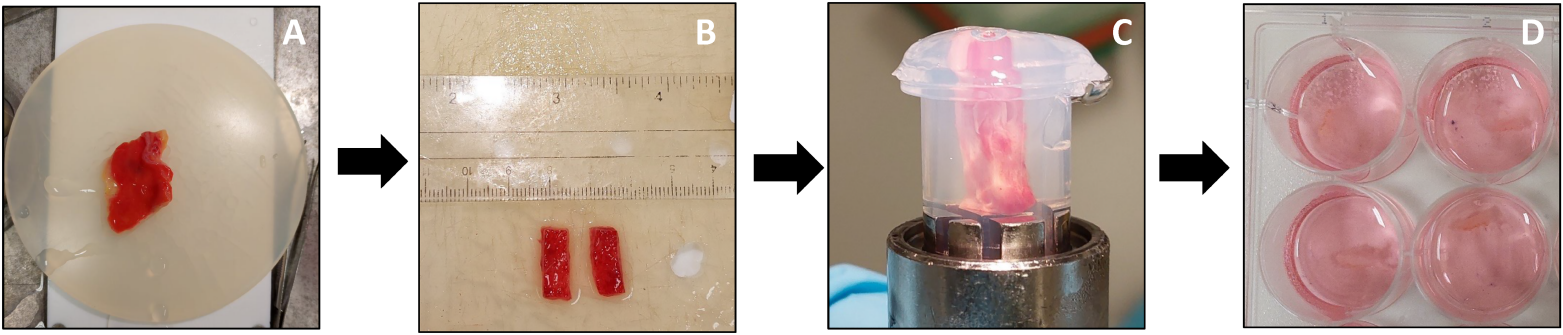
Preparation and slicing of human PCGS. The gallbladder tissue (**A**) was first cleaned and fat were removed. Next, the gallbladder was cut into small patches (**B**). The small patch was then placed in 3% low-gelling-temperature agarose. After solidification of the agarose (**C**), the core was removed from the core holder (**D**) and placed in the Krumdieck tissue slicer to prepare the PCGS. After slicing, the PCGS were transferred into 12 well culture plates filled with culture medium.

Similar to the preparation of the cystic duct cores, the gallbladder tissue was first cleared from as much of the surrounding fat and loose connective tissue as possible. The gallbladder was then cut into small patches (0.5 cm in width, 1.5 cm in length). A smaller patch (0.5 cm in width, 0.5 cm in length) was preserved in 4% formalin for morphology. A patch was then placed in the metal core holder, which was then filled quickly with 3% low-gelling-temperature agarose type VII (Sigma-Aldrich, Zwijndrecht, The Netherlands) at a temperature of 37°C. After solidification of the agarose, the core was ready to be used for slicing and was placed in the Krumdieck slicer. An overview of the procedure is shown in Figure 2.

#### 2.2.3. Slicing of the tissue cores

PCCDS and PCGS were prepared using the Krumdieck tissue slicer. The Krumdieck tissue slicer was filled with ice-cold Krebs-Henseleit buffer with a pH of 7.4, which was supplemented with 25 mM D-glucose (Merck), 25 mM NaHCO_3_ (Merck) and 10 mM HEPES (MP Biomedicals, Aurora, USA) and saturated with carbogen (5% O_2_/5% CO_2_). The arm speed was set at the lowest speed, whilst the blade speed was set at the highest speed. The wet weight of the slices was measured at the start of the slicing and the slicer was adjusted accordingly. The wet weight is generally used as an indicator of thickness of the slices. The wet weight of PCGS was 5–6 mg. As the appearance and diameter of the cystic duct specimens varies between patients, the wet weight of the PCGS was used to adjust the slicer for the PCCDS. The slicer was set to a wet weight of 10 mg for the PCGS and this setting was then used to prepare the PCCDS. At this setting, it was possible to prepare intact PCCDS. Slices were collected and stored on ice in UW solution until transfer into culture plates. Three slices were immediately preserved in 4% formalin.

### 2.3. Incubation of the PCCDS and PCGS

The PCCDS and PCGS were incubated in 12-well culture plates, in which each well contained 1.3 ml pre-warmed (37°C) and oxygenated cultured medium. The standard culture medium was composed of William's Medium E (WME) with Glutamax (Gibco, Invitrogen, Paisley, Scotland) supplemented with 25 mM d-glucose and 2 μg/ml ciprofloxacin, as reported in previous human PCTS studies (1, 10). Thermal pads were used during the transfer of the slices into the plates. The culture plates with slices were placed in an incubator (37°C; 80% O_2_; 5% CO_2_), which was gently shaking (90 times/minute) for 24 h. After the 24 and/or 48 h incubation period, slices were harvested to determine the ATP and protein content and study their morphology.

### 2.4. ATP content

For each ATP sample (3 samples per condition), one slice was collected in a collection tube filled with 0.5 ml sonification solution (70% ethanol and 2 mM EDTA) and mini-beads, immediately snap-frozen in liquid nitrogen and stored at −80°C. For ATP determination, samples were placed in a Minibead-beater for homogenization (2 rounds of 45 s) and subsequently centrifuged for 5 min at 13.000 rounds per minute. ATP content was measured in the supernatant using the ATP Bioluminescence Kit (Roche Diagnostics, Mannheim, Germany) in the same manner as previously described (1). The precipitate was left to dry in a 37°C stove for 1 day or at room temperature for 3 days and subsequently used for protein determination. The ATP content (pmol) was normalized to the total amount of protein (µg), which was measured using the RC DC Protein Assay (Bio-Rad, Munich, Germany).

### 2.5. Morphology

For each morphology sample (1 per condition), three slices were fixated in 4% formalin, stored at 4°C for 24 h and then transferred to 70% ethanol. After dehydration with ethanol (in increasing concentrations), slices were embedded in paraffin and then cut into 4 µm sections. In preparation for haematoxylin and eosin staining (H&E), sections were deparaffinized with xylene and rehydrated with ethanol (in decreasing concentrations). After staining, sections were dehydrated again.

Blinded morphological assessment of the tissue sections was performed by an experienced pathologist. The tissue sections were assessed using a newly developed scoring protocol (Table 1). This scoring protocol was based on several morphological features of the slices (i.e., type and amount of inflammation, integrity of surface epithelium, presence of epithelial crypts, re-epithelialization after incubation, viability and in case of PCCDS the presence of peribiliary glands). The scoring item viability was defined as preservation of tissue morphology. These features are considered to be of importance for the feasibility of the model for research into cholangiopathies. For each item of the protocol, a score was given. A higher score correlated to a poorer state of the slice regarding that specific item. A total score was calculated and compared to the ATP/protein content with the aim to assess whether there was a correlation between both indicators of viability.

**Table 1:** Scoring protocol for H&E stained sections of the PCGS and PCCDS.

Items for scoring	Category 0	Category 1	Category 2	Category 3
Crypts of the epithelium (% present)	0 (100%)	1 (66%)	2 (33%)	3 (0%)
Inflammation 1 (type)	0 (none)	1 (chronic)	2 (active/active and chronic)	
Inflammation 2 (amount)	0 (none)	1 (slight)	2 (moderate)	3 (severe)
Viability (overal, %)	0 (100%)	1 (66% viable, 33% non-viable)	2 (33% viable, 66% non-viable)	3 (100% viable)
Surface epithelium				
Before incubation: amount of damage	0 (none)	1 (slightly erosive)	2 (moderately erosive)	3 (severly erosive)
After incubation: formation of a new cell layer at the epithelial side (% of the slice)	0 (100%)	1 (66%)	2 (33%)	3 (0%)
PCCDS only				
Peribiliary glands	0 (present, viable	1 (present, non-viable)	2 (absent)	
Total score				
PCGS = 17				
PCCDS = 19				

The type of inflammation was based on the presence of lymphocytes and plasma cells (indicating chronic inflammation) and/or the presence of neutrophil granulocytes (indicating active inflammation).

In addition to H&E staining, immunohistochemical staining for pan Cytokeratin (Cytokeratin, Multi (AE1/AE3) Bond RTU Primary, Leica Biosystems; Mouse anti Pan Cytokeratin, clone BS5 (Monoclonal), Monosan) was also performed. Quantification of the pan Cytokeratin staining was performed using QuPath (version QuPath-0.4.3), which is an open source software program for bioimage analysis (see Supplementary Material for further details) (12).

### 2.6. Statistical analysis

GraphPad Prism 9.4.1 was used for statistical analyses of the data. The paired samples t-test was used to analyze whether a significant change occurred in ATP, protein, ATP/protein content and positive cell count of the pan Cytokeratin staining after 24 and 48 h incubation compared to 0 h. The mean of three replicate slices per cystic duct/gallbladder specimen (technical replicates) was used for the analyses.

To assess the correlation between ATP/protein content and the morphology score, the Pearson correlation coefficient (*r*) was calculated. Morphology score was considered a continuous variable as it is a sum of multiple scores (which are quantitative), however the exact total score does not correlate to a specific category/does not carry any meaning. To determine whether statistically significant differences were present between the morphology scores of the different time points, the Repeated Measurements one-way ANOVA for the PCGS and paired samples t-test for the PCCDS were performed. To compare the results of several scoring items (i.e., epithelial crypts, re-epithelialization and viability) between the time points, the Wilcoxon matched-pairs signed rank test was used. The alpha level for each statistical analysis was set at.05.

## 3. Results

### 3.1. Cystic duct and gallbladder specimens used for preparation of the PCCDS and PCGS

Tissue slices could be prepared from cystic duct (*n* = 9) and gallbladder (*n* = 11) specimens and were used to study the viability after different incubation periods. One cystic duct and three gallbladders were excluded based on very low ATP/protein content in combination with poor, mostly non-viable morphology after incubation. Results from the remaining cystic ducts (*n* = 8) and gallbladders (*n* = 8) will be shown below. Patient characteristics are shown in Table 2.

**Table 2:** Patient characteristics of the cystic duct and gallbladder specimens used for preparation of the PCCDS and PCGS.

Sample code gallbladder/cystic duct	Age (years)
23	Unknown
25	53
26	30
27	76
38	28
39	37
40	Unknown
45	Unknown
51	Unknown
52	37

Mean age in years was 43.5 ± 7.42.

### 3.2. Viability of PCCDS and PCGS

#### 3.2.1. ATP/protein content

**Figure 3:**
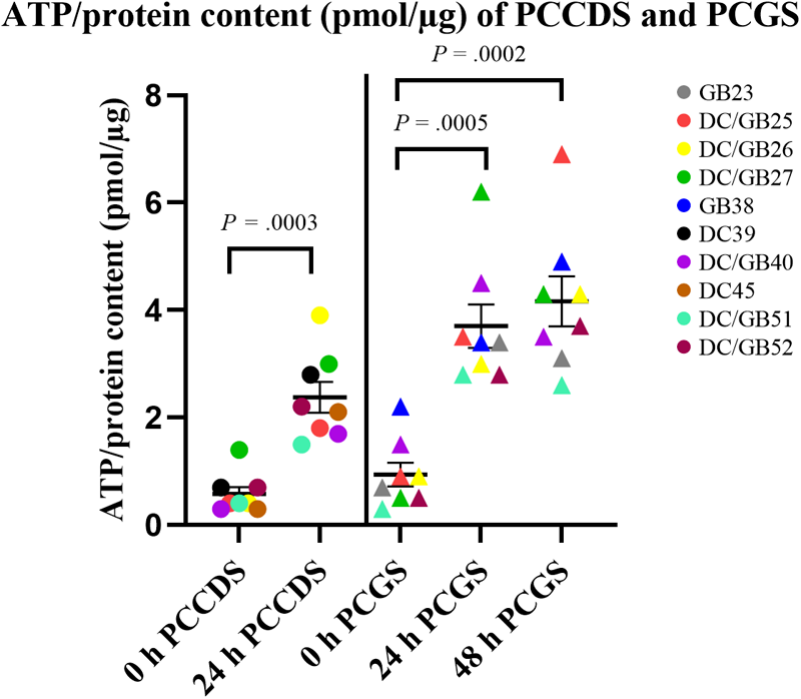
Viability of PCCDS and PCGS at 0 h and after 24 and 48 h incubation as assessed by the ATP/protein content. The cystic duct and gallbladder specimens from one patient were coded with the same color. *P*-values denote statistical significance.

The ATP/protein content of the PCCDS increased significantly from 0.58 ± 0.13 pmol/µg at 0 h to 2.4 ± 0.29 pmol/µg after 24 h incubation (*P* = .0003) (Table 3). A comparable increase from 0.94 ± 0.22 pmol/µg at 0 h to 3.7 ± 0.41 pmol/µg after 24 h (*P* = .0005) and 4.2 ± 0.47 pmol/µg after 48 h (*P* = .0002) was observed in the PCGS (Table 3, Figure 3).

**Table 3:** Mean ± standard error of mean (SEM) of ATP, protein and ATP/protein values of PCCDS and PCGS at 0 h and after 24 and 48 h incubation.

	PCCDS (*n* = 8)				PCGS (*n* = 8)		
	0 h (3 slices) (*n* = 5)	0 h (1 slice) (*n* = 5)	0 h combined (*n* = 8)	24 h (1 slice)	0 h (3 slices)	24 h (1 slice)	48 h (1 slice)
ATP (pmol)	1,393 ± 164	552 ± 161		3,781 ± 566	1,135 ± 212	1,693 ± 237	1,916 ± 260
Protein (µg)	2,872 ± 762	1,211 ± 239		1,643 ± 157	1,315 ± 141	498 ± 57	469 ± 40
ATP/protein (pmol/µg)	0.64 ± 0.20	0.48 ± 0.09	0.58 ± 0.13	2.4 ± 0.29*	0.94 ± 0.22	3.7 ± 0.41*	4.2 ± 0.47*

ATP/protein content increased significantly after the 24 and 48 h incubation period compared to 0 h.

* *P* < .05.

The absolute ATP (pmol), protein (µg) and ATP/protein (pmol/µg) content of the PCGS did not change significantly between the 24 and 48 h incubation period (*P* = .40, *P* = .52 and *P* = .46). This shows that the viability was not affected by a prolonged incubation period of 48 h compared to 24 h.

ATP/protein values indicate that the cystic duct and gallbladder tissue and the subsequently prepared PCCDS and PCGS remained viable during processing, including storage, transport and slicing procedure.

#### 3.2.2. Morphology: H&E staining

**Figure 4:**
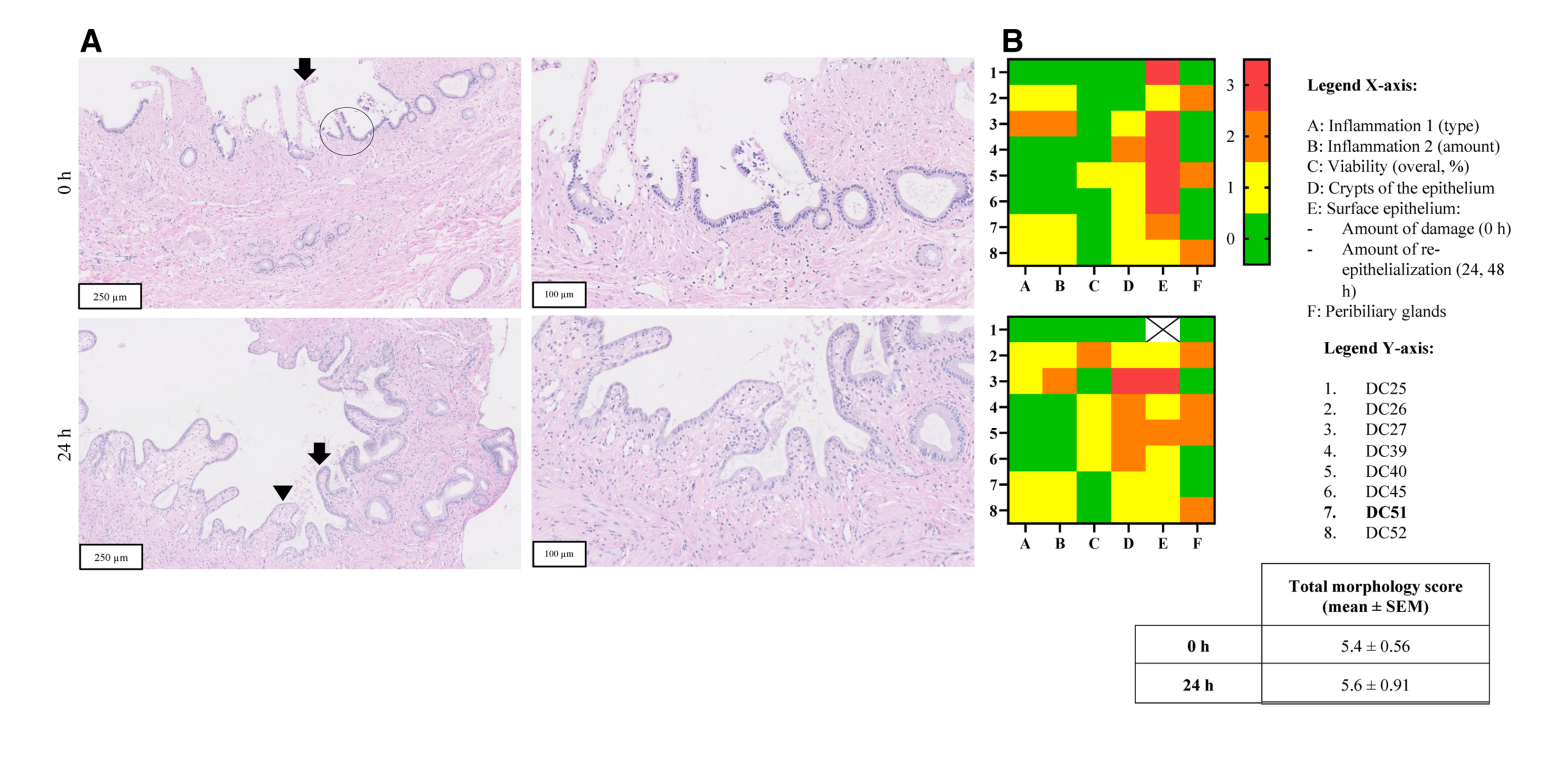
Panel **A** shows H&E stained sections of representative PCCDS before and after incubation from one patient (sample code: DC51) (magnification 10 x, 250 µm and 20 × 100 µm). The arrow points towards the epithelium, which shows erosion. A viable crypt of the epithelium is encircled. The arrow points towards viable epithelium and arrowhead indicates a new epithelial cell layer. In terms of morphological scoring of these PCCDS, the following scores were attributed to the slices at 0 h: slight chronic inflammation (score 1), 100% viability (score 0), 66% of epithelium contained crypts (score 1), moderately erosive surface epithelium (score 2) and viable PBG present (score 0). The other scores of these PCCDS and PCGS can be found in row 7 of the heatmaps in Figure 5 (sample codes DC51). Panel **B** shows heatmaps of morphological scoring of the PCCDS. Scoring items are shown on the x-axis and the different PCCDS samples on the y-axis. Sample codes of the cystic ducts used to prepare the slices can be found in the y-axis legend. Scores are represented by different colors, whereby 0 = green, 1 = yellow, 2 = orange and 3 = red.

**Figure 5:**
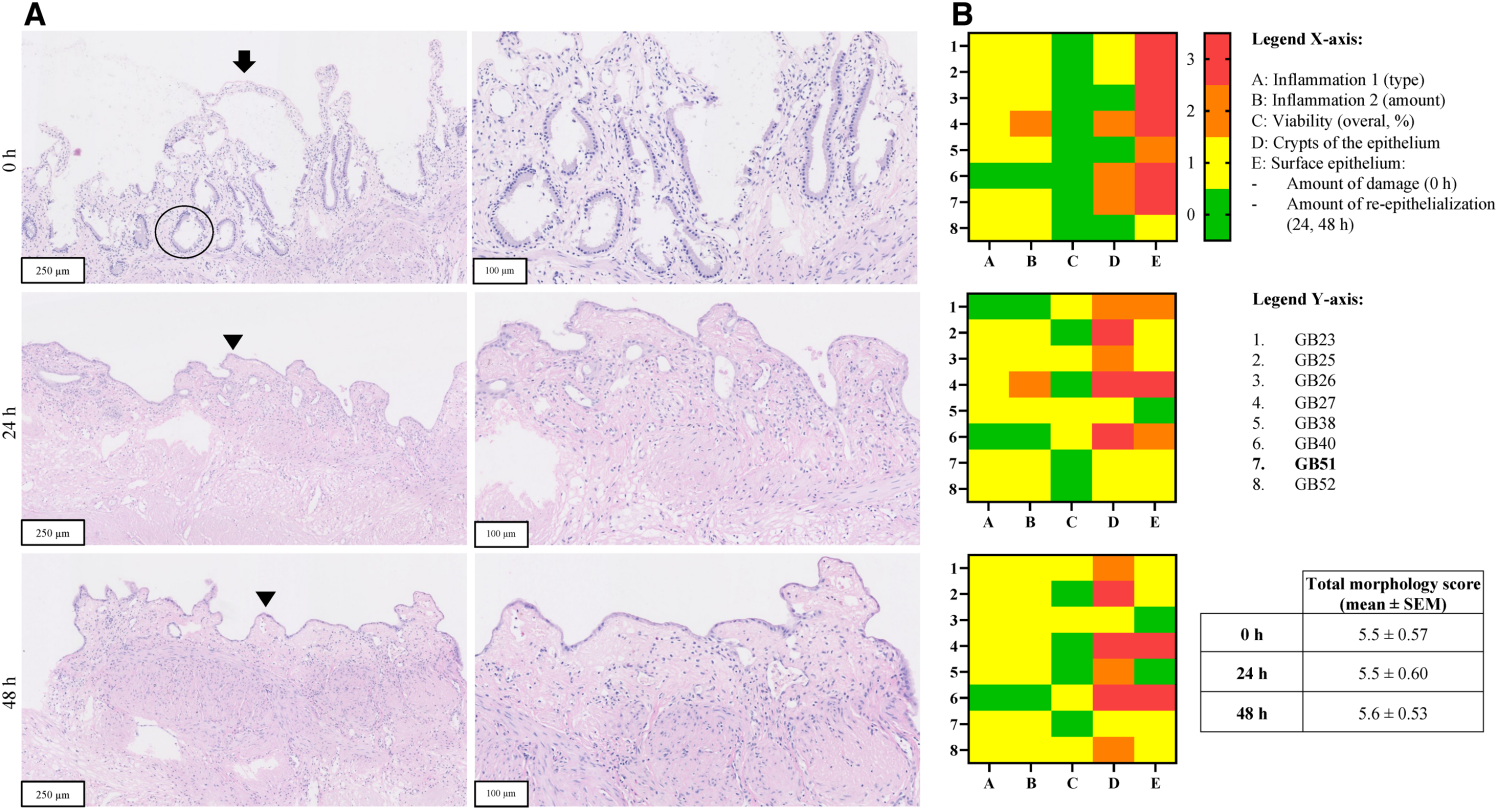
Panel **A** shows H&E stained sections of representative PCGS before and after incubation from one patient (sample code: GB51) (magnification 10 x, 250 µm and 20 × 100 µm). The arrow points towards the epithelium, which shows erosion. A viable crypt of the epithelium is encircled. The arrow points towards viable epithelium and arrowhead indicates a new epithelial cell layer. Panel **B** shows heatmaps of morphological scoring of the PCCDS. Scoring items are shown on the *x*-axis and the different PCGS samples on the *y*-axis. Sample codes of the gallbladders used to prepare the slices can be found in the *y*-axis legend. Scores are represented by different colors, whereby 0 = green, 1 = yellow, 2 = orange and 3 = red.

H&E staining was performed on the tissue sections of the PCCDS (*n* = 8) and PCGS (*n* = 8) (Figures 4, 5).

##### 3.2.2.1. PCCDS sections

H&E stained sections of PCCDS collected at 0 h and after 24 h incubation were assessed using a scoring protocol and the results are shown in Figure 4. Each sample consisted of three slices, of which the average was taken into consideration for the scoring. Results from the scoring of the PCCDS sections are shown in the heatmaps in Figure 4 as well. At 0 h, all samples were viable for at least 66%. After 24 h incubation, seven out of eight samples remained viable. One sample decreased in viability and was viable for only 33%. Viability did not change significantly with incubation compared to 0 h (*P* = .25). Chronic inflammation was present in half of the samples at 0 and 24 h. In one of these samples, active inflammation was also seen at 0 h, but not at 24 h. The amount was inflammation was slight in most samples except for one. PBG were present in five out of eight samples at 0 h and in four of the eight samples after 24 h. PBG appeared viable in all samples. Before incubation, the surface epithelium was erosive in all samples, but the degree of erosion varied. After incubation, re-epithelialization occurred in seven out of eight samples. The sample without epithelialization retained its normal epithelial layer. This new epithelial layer covered 66% of the slice in all but one sample in which it covered 33%. Crypts were present in at least 66% of the epithelium in 7 out of 8 samples at 0 h. After incubation, the amount of crypts appeared to decrease, however not significantly (*P* = .13). The overall morphology score did not change significantly between the time points (*P *= .65).

##### 3.2.2.2. PCGS sections

H&E stained tissue sections of PCGS collected at 0, 24 and 48 h were assessed in a similar manner as the PCCDS using the scoring protocol (Figure 5). Results from the scoring of the PCGS sections are shown in the heatmaps in Figure 5 as well. Before incubation, all samples were viable. Four samples showed similar viability after 24 h incubation, whereas the other four decreased in viability to 66%. Viability did not change with 48 h incubation compared to 24 h. Viability did not change significantly after 24 and 48 h incubation compared to 0 h (both *P* = .13). Nearly all samples showed signs of slight, chronic inflammation at each time point. At 0 h, the surface epithelium was severely erosive in most samples except for two, which showed a lesser degree of erosion. After 24 h incubation, re-epithelialization occurred in seven samples and covered between 33% and 100% of the slice. The degree of re-epithelization did not change significantly after 48 h compared to 24 h (*P *> .99). Epithelial crypts were present in at least 33% of the epithelium at 0 h. After incubation, three samples did not show epithelial crypts anymore. The amount of crypts tended to decrease after 24 h (*P* = .05) and decreased significantly after 48 h incubation (*P* = .047) when compared to 0 h. The overall morphology score did not change significantly between the time points (*P* = .94).

#### 3.2.3. Correlation between ATP/protein content and morphology

**Figure 6:**
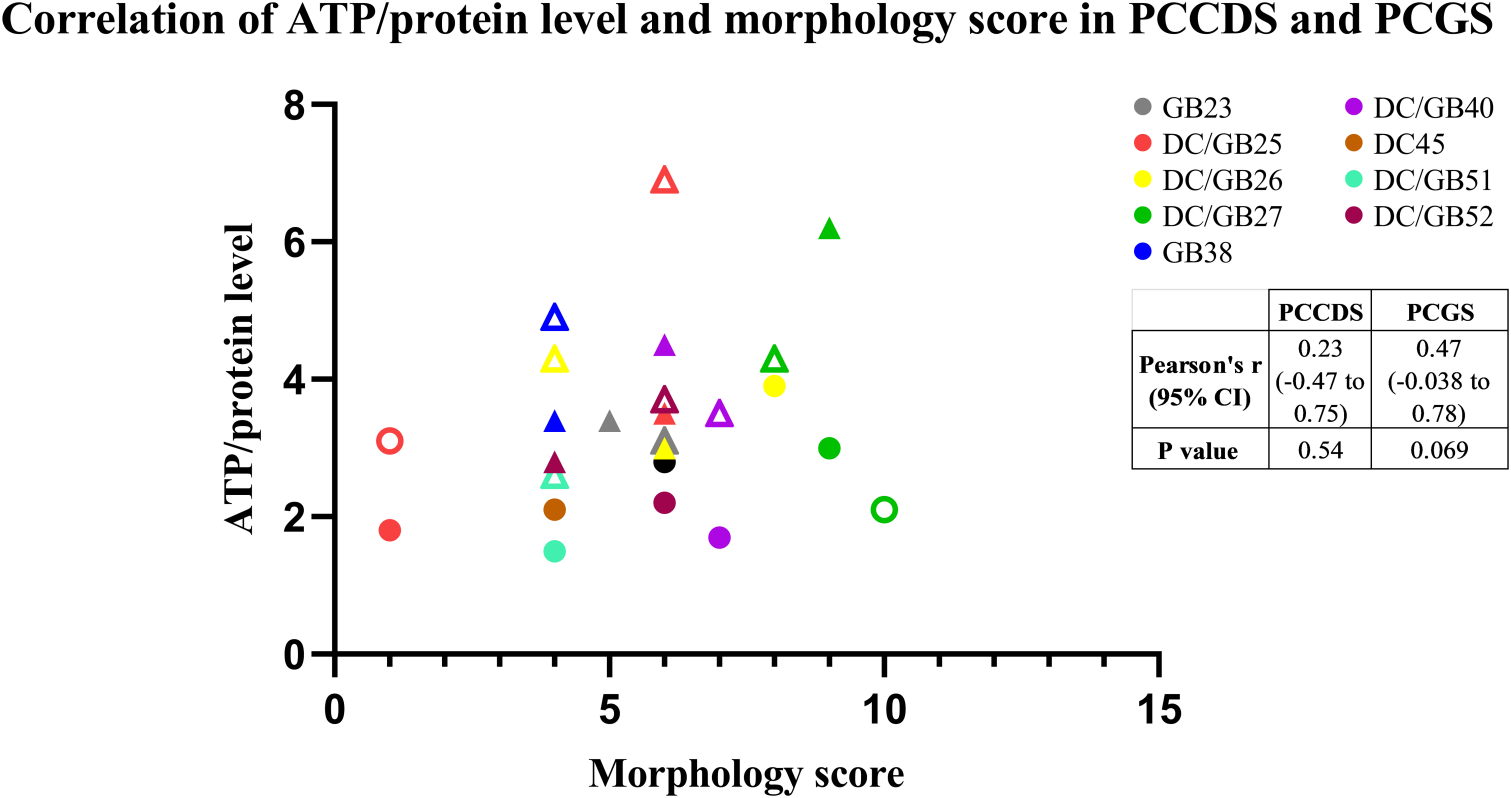
Correlation between morphology score and ATP/protein level of the PCCDS and PCGS. The results are shown for each cystic duct or gallbladder separately (*n* = 8). Solid circles represent samples after 24 h incubation and transparent samples represent 48 h incubation samples.

The correlation between ATP/protein content and morphology indicated by the morphology score was assessed (Figure 5). The Pearson correlation coefficient was 0.23 (*P *= .54) for the PCCDS and 0.47 (*P *= .07) for the PCGS, which does not show a correlation (Figure 6).

#### 3.2.4. Immunohistochemical staining of PCCDS and PCGS sections

**Figure 7:**
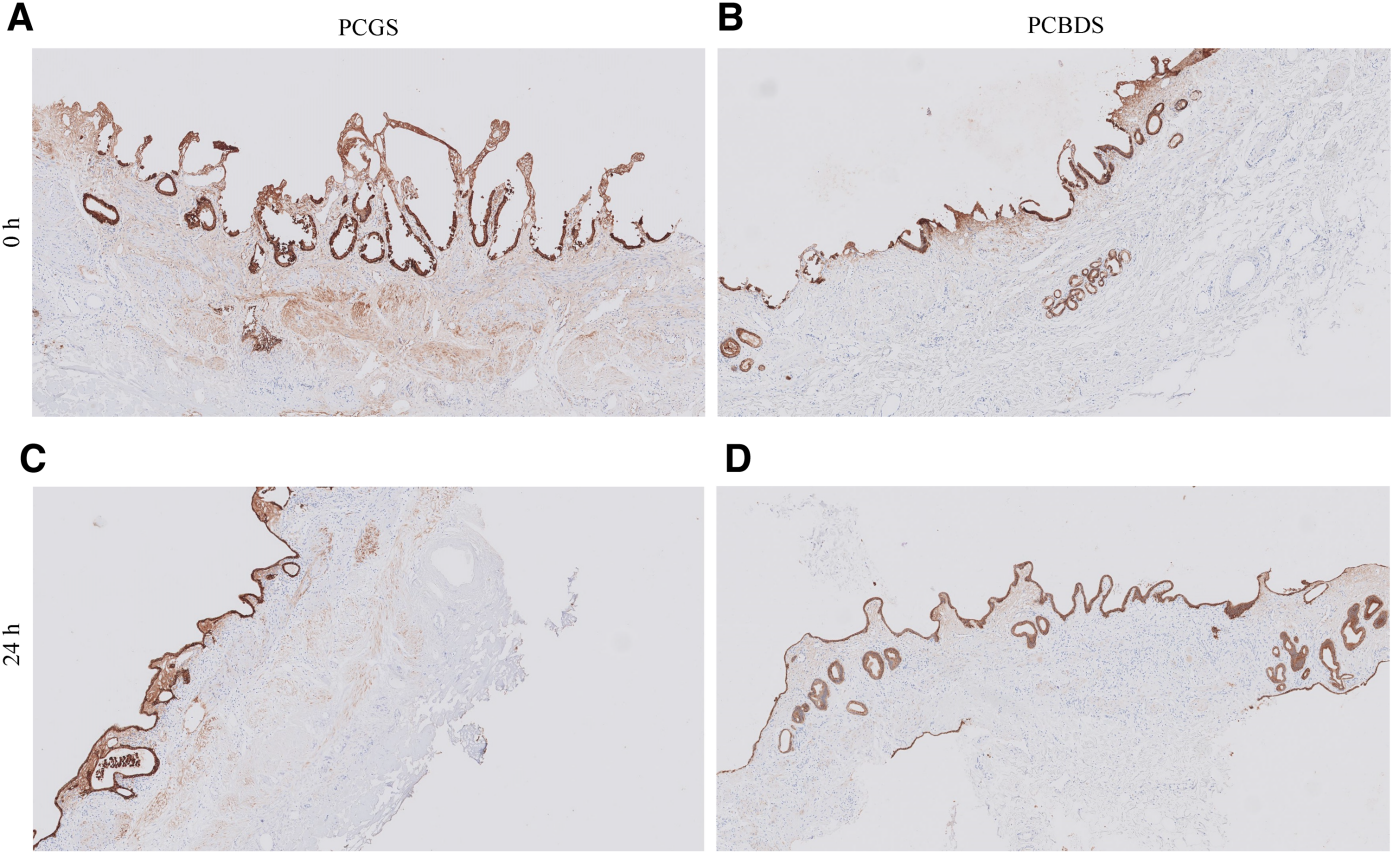
Immunohistochemical staining for pan cytokeratin of PCGS and PCCDS. Before incubation, the surface epithelium of the PCGS and PCCDS stains positive (images **A,B**). After incubation, the new cell layer at the side of the epithelium also stains positive (images **C,D**). Positive staining indicates the presence of epithelial cells.

**Figure 8:**
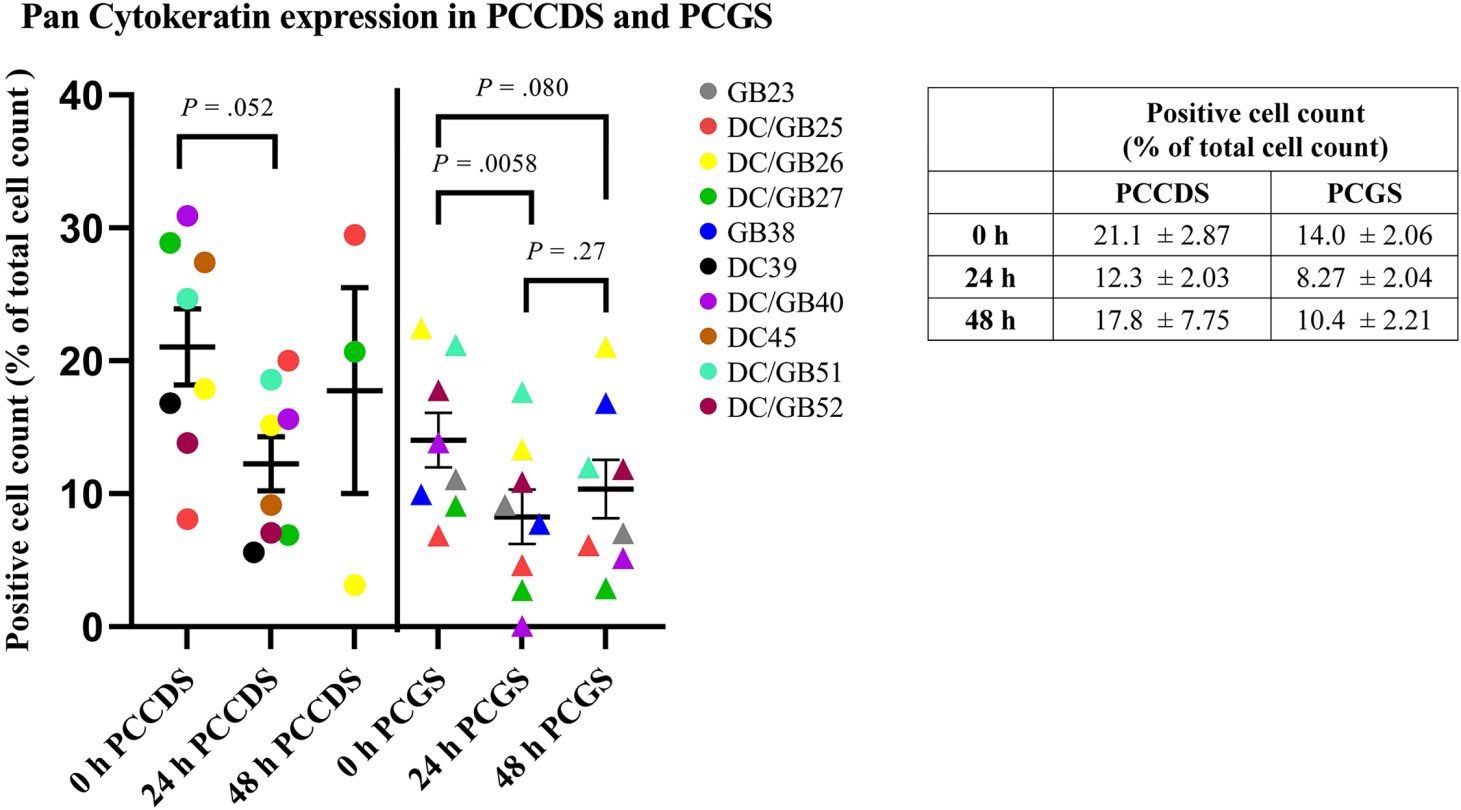
Quantification of pan cytokeratin staining of PCCDS and PCGS. The figure shows the percentage of positive cells for each cystic duct and gallbladder separately. In the table, the exact values are shown.

Tissue sections from all PCCDS and PCGS samples were immunohistochemically stained for pan Cytokeratin (Figure 7). At 0 h, 21.1% of the cells of the PCCDS stained positive for pan Cytokeratin (Figure 8). After 24 h incubation, a decreasing trend was observed, in which 12.3% of the cells were positive (*P* = .052). In the PCGS, 14.0% of the cells were pan Cytokeratin positive at 0 h, which decreased to 8.27% after 24 h (*P* = .0058) and 10.4% after 48 h (*P* = .080) (Figure 8). The apparent increase after 48 h compared to 24 h was not significant (*P* = .27). The new cell layer described in the semi-quantitative analysis stained positive in all samples after incubation.

## 4. Discussion

The primary aim of this study was to develop and optimize precision-cut cystic duct slices (PCCDS) and precision-cut gallbladder slices (PCGS) as an *ex vivo* model, as this model may provide novel opportunities for research in the field of cholangiopathies such as biliary atresia. As of yet, the PCCDS model has been used only once (10) and the full potential of this model is yet to be discovered. Results of this study have shown that viability, assessed by the ATP/protein content and morphology, of the PCCDS and PCGS could be maintained over the 24 and 48 h incubation period respectively. ATP/protein content in combination with morphology have been shown to be reliable indicators of viability of precision-cut liver slices and other PCTS models (1, 13) and were therefore also used as viability markers for the PCCDS and PCGS.

Firstly, with regard to incubation of the PCCDS and PCGS, it was shown that the ATP/protein content was very low at 0 h, but significantly increased during the 24 and 48 h incubation period. This is in accordance with PCTS from other organs or species (1, 14). The observed low ATP/protein content of the slices at 0 h can be explained by the fact that the tissue has been stored in cold UW organ preservation solution for approximately 24 h and processed into slices at similar temperatures in order to prevent tissue degradation. At low temperatures, the enzymes responsible for synthesis of ATP (ATP synthases) are inactivated and therefore a low ATP/protein content is found in the PCCDS and PCGS at 0 h. Thus, ATP/protein content is not representative of tissue viability at 0 h, only after reactivation of these ATP enzymes during incubation, the ATP/protein content becomes indicative of tissue viability. During incubation of the slices at 37°C in pre-warmed and oxygenated culture medium, the ATP synthases will be reactivated and hence the ATP content will increase. Unfortunately, it is impossible to compare our results to other PCTS models due to organ-specific differences in terms of cell types. The level of ATP production differs per cell type and different organs are composed of different cell types. For example, hepatocytes have a great capacity to produce ATP and they are a predominant cell type in the liver. Hence, the ATP content of precision-cut liver slices (PCLS) can vary between 2 and 14 pmol/µg, which is higher than the ATP content of the PCCDS and PCGS (1). Given the above-mentioned differences between organs, the ATP content of a specific PCTS model can only by reliably related and compared to previously obtained values in the same model.

In total, 11 gallbladders and 9 cystic ducts were used to prepare slices, of which only three gallbladders and one cystic duct were excluded. These PCGS and PCCDS did not show an increase in ATP content and had a poor, mostly non-viable morphology after incubation. This might have been due to the surgical procedure or due to delayed transfer of the tissue to UW organ preservation solution after resection. Transfer of the resection specimen to the pathology department usually takes place only after closure of the skin, which implies that the gallbladder will be stored in the OR in room temperature for some 15 min, which might affect viability to some extent. Before transfer, the tissue is not actively cooled and metabolic processes will continue despite the ischemia. As demonstrated in organ transplantation studies, this period of sustained metabolism is detrimental to tissue viability due to depletion of ATP and formation of reactive oxygen species (ROS) during re-oxygenation (15). UW solution contains several components to limit these harmful processes, such as allopurinol and glutathione (11, 16). The non-viable cystic duct derived from the same patient as one of the non-viable gallbladders, which is in support of either proposed explanation. Nonetheless, as this is a very standardized procedure, we do not expect a large variation between cases. This was also demonstrated by the fact that he majority of gallbladders and cystic ducts used for this study were viable.

In addition to the ATP/protein results, morphological assessment of the PCCDS also showed viable tissue. Of note, no major differences between the morphology of tissue sections at 0 h and after 24 or 48 h incubation could be observed. Most PCGS showed signs of moderate chronic inflammation. This can be explained by the fact that only gallbladders from patients undergoing a cholecystectomy for gallstone disease were used. As previously mentioned, ATP/protein content was low at 0 h, which can be attributed inactivation of the necessary enzymes due to cold preservation of the tissue and slices. Our finding that morphology showed viable tissue at 0 and 24 h supports the fact that ATP/protein content at 0 h is not representative of viability. Interestingly, PCCDS and PCGS incubated for 24 and 48 h in the standard culture medium showed clear alterations in the epithelial structure. At this moment, two possible hypotheses could be formulated about this change in morphology of the epithelium. Firstly, it could be due to flattening of the epithelial layer, whereby the simple columnar epithelial cells transformed into smaller, flattened epithelial cells. Possible causes of columnar cell flattening include enlargement of the apical (facing external environment) and basal (facing internal environment) surfaces or loss of adhesion molecules at the lateral surfaces of epithelial cells (17). Similar changes in epithelium were also observed in rat and mouse precision-cut intestinal slices (PCIS) (18). On the other hand, it could also be a new layer of cells growing at the epithelial side on the areas that were denuded from epithelium. In support of this is the fact that the epithelium of the slices was mostly severely erosive at 0 h and did not have such a cell layer yet. Interestingly, the amount of epithelial crypts decreased with incubation. This could support the hypothesis that a new epithelial layer grows from within the crypts during incubation. Immunohistochemical staining of the slices showed that this new cell layer expressed cytokeratin, which confirmed that these cells were indeed of epithelial origin. This fits well with the second and presumably more plausible hypothesis, especially given the lack of epithelium before incubation and the decline in number of crypts afterwards. In the next study, we will focus on stimulation of this re-epithelialization process using growth factor and cytokines that stimulate the proliferation and differentiation of stem/progenitor cells residing in the peribiliary glands of the cystic duct and epithelial crypts of the gallbladder. Quantification of the pan Cytokeratin showed that the percentage of positive cells significantly decreased after 24 h incubation in PCGS and showed a similar trend in PCCDS. In the semi-quantitative analysis, it was observed that the surface epithelium at 0 h was often already erosive, especially in the PCGS. These damaged epithelial cells did stain positive, but might have degraded in the 24 h incubation period, which could explain the decrease in positive cell count. The positive cell count remained stable between the 24 and 48 h time points, which supports this hypothesis.

Next, we have demonstrated that the ATP content did not correlate with the degree of morphological integrity of viable PCCDS and PCGS. This might be due to the selection of scoring criteria, which, in addition to viability, also focused on other morphological features deemed important for the functionality of the model. Another explanation could be that the cell type primarily responsible for ATP production is not scored on morphology. For example, B-lymphocytes might contribute to the total ATP content and is scored as part of inflammation. In this case, a sample with a higher and thus poorer score regarding inflammation may also have a higher ATP content, as more ATP producing B-lymphocytes are present. Another cell type that might also be an important contributor to the ATP content are smooth muscle cells. Smooth muscle is more abundantly present in the gallbladder compared to the cystic duct (19), which might explain the difference in ATP content. As the smooth muscle layer is not scored in our morphological scoring system, this might result in the observed lack of correlation. The correlation between ATP content and morphology appeared stronger in the PCGS compared to the PCCDS, however neither correlation was significant. This might be related to differences in ATP producing cell types between the two, for example the aforementioned B-lymphocytes and muscle cells which are more profusely present in PCGS. For statistical testing, the morphology score was considered a continuous variable, as it is a sum of multiple scores (which are quantitative), however the exact total score does not correlate to a specific category/does not carry any meaning. On the other hand, the morphology score could also be regarded as semi-quantitative as it contains integers and values are within a certain range. Despite the fact that some caution is therefore warranted when interpreting the results of the Pearson's *r* correlation coefficient itself, it can still be deduced from Figure 6 that there is no clear correlation between ATP content and morphology score. Using our scoring system, ATP content is not a good indicator of morphological integrity of PCCDS and PCGS, in contrast to studies using PCTS from other organs. Indeed, Bigaeva et al. (18) showed that the degree of morphological integrity correlated with ATP content in PCIS, whereby a decline in ATP was accompanied by structural alterations in the slices. In the above-mentioned study, incubation of the PCIS with organoid medium, which is enriched with growth factors (epithelial growth factor (EGF), Noggin and R-spondin) resulted in a longer lifespan (up to 96 h) of the slices and lead to an improvement in slice morphology of mouse PCIS, including improved preservation of epithelial integrity (18). A similar improvement after incubation with organoid medium also occurred in human PCIS (20). It would be interesting to investigate whether incubation in different culture media, possibly also supplemented with growth factors, can improve the lifespan of the PCCDS and PCGS, both in terms of ATP/protein content and morphology.

Noteworthy for the preparation of the PCCDS were the variations in terms of structure and amount of cystic duct tissue available for slicing. The cystic duct remnant sometimes still consisted of a tube-like structure of 2–3 cm and at other times, only 1.5 cm or less was left. In this study, it was possible to obtain enough PCCDS for each cystic duct (15 PCCDS on average). However, it might impose limitations on future studies regarding the number subsequent analyses (e.g., Western Blot, qPCR) that can be performed on PCCDS from one cystic duct. Notwithstanding the limited number of slices per cystic duct, the supply of cystic duct tissue is virtually unlimited, which provides ample opportunity for further studies. Moreover, the number of PCGS that can be prepared from the provided amount of gallbladder tissue will probably suffice for future studies that require additional analyses.

A limitation of this study was that the exact slice thickness of the PCCDS and PCGS was not determined. Wet weight is normally used as an indicator for thickness of the slices, which in turn correlates to viability, as it determines the amount of oxygen and nutrient supply to the cells of the slices. Thickness, represented by the wet weight, should therefore be carefully determined and be set at the same for every slice. The wet weight of the PCGS was set at 5–6 mg, which is similar to the wet weight of PCTS from other organs (1). Due to variations in the diameter of the cystic duct, the wet weight of the PCCDS could not be used as an accurate indicator of thickness. Therefore, we decided to use the weight of the PCGS as a reference for the PCCDS, as explained in the methods section. With the Krumdieck slicer at the setting of 5–6 mg wet weight of PCGS, it was impossible to create intact PCCDS. Therefore, we decided to set the Krumdieck slicer at 10 mg wet weight of PCGS. Despite the fact that the PCCDS and PCGS were viable and the inner cell layers did not show necrosis, which indicates an appropriate thickness, we did not measure the exact thickness. For future use of the PCCDS and PCGS, it might be valuable to determine this to ensure appropriate slice thickness.

One of the main advantages of this human PCCDS/PCGS model over the currently used models for studying cholangiopathies (i.e., cholangiocyte cell cultures, bile duct ligation mouse models, transgenic mouse models, organoids from induced pluripotent stem cells of patients) (21) is that it preserves the natural environment of cholangiocytes and their interactions with other cell types, thus resembling *in vivo* physiological circumstances. Due to this resemblance, findings can be translated and applied to the *in vivo* situation. *In vivo* cell cultures neither represent the multicellularity nor the three-dimensional structure of an organ/tissue and loss of polarization may occur in certain cell types. A great advantage of human PCCDS/PCGS over animal models is that human cystic duct and gallbladder tissue can be studied, which is more representative of the *in vivo* situation. Animal models often do not fully reflect the human *in vivo* conditions, which may impair translation of findings to humans. In addition, costs in terms of both animal lives and money are often high. As opposed to organoids, which consist of clusters of cells growing in different directions, the natural tissue structure of the cystic duct and therefore environment of cholangiocytes is maintained in PCCDS. A possible purpose of this model in cholangiopathy research could be to study effects of compounds that may be implicated in the pathogenesis on the bile duct/gallbladder, e.g., the identified toxin biliatresone that may play a role in biliary atresia (22). As PCTS are commonly used to study drug efficacy, metabolism and toxicity (6, 23, 24), PCCDS/PCGS could also be used the study the effects of novel drugs for treatment of cholangiopathies. For future use of PCCDS and PCGS as a multicellular model, it would be worthwhile to further study the different cell types present in the walls of the cystic duct and gallbladder and obtain details regarding the phenotypes of the different cellular components.

As for every model, there are certain limitations to the PCCDS/PCGS model, including the lack of involvement of other organs and the immune system and absence of blood flow. Nevertheless, as long as these limitations are kept in mind when translating findings to the *in vivo* situation, it should not impair the use of PCCDS/PCGS in studying for example the pathogenesis of diseases. Another limitation is that the morphology of the PCCDS and PCGS could not be compared to the morphology of the tissue immediately after surgery, so before overnight storage. In future studies, it would be valuable to collect these samples as well, so that possible changes after overnight storage could be identified. Since morphological assessment was based on H&E staining only, future studies should also include tissue evaluation using TUNEL-staining or immunohistochemical staining for cell death markers to definitely rule out apoptosis.

In conclusion, in this study a method for preparation and culturing of PCCDS and PCGS from cholecystectomy tissue was developed, which was shown to be successful as the slices remained viable for an incubation period of at least 24 h. As the source of the tissue was the routinely performed cholecystectomy procedure, tissue can be easily acquired will always be available for future studies. After further optimization of the technique, PCCDS may serve as a novel model for studying cholangiopathies *in vitro*. It may be a valuable model for research regarding pathogenesis and aetiology of cholangiopathies as the complete network of cells and their interactions are preserved in the slices, which makes them more representative of *in vivo* processes compared to the currently available *in vitro* cell culture models.

## Data Availability

The raw data supporting the conclusions of this article will be made available by the authors, without undue reservation.
