# Supplementary material for: Human precision-cut cystic duct and gallbladder slices: a novel method for studying cholangiopathies
Source: Front Pediatr. 2023 Jul 17;11:1058319. doi: 10.3389/fped.2023.1058319 (PMC10387522; doi:10.3389/fped.2023.1058319)
Supplement: Supplementary file 1 [file Table1.docx]

**Supplementary information**

**Quantification of pan Cytokeratin staining**

QuPath is an open source bioimage analysis software program, which produces fast, accurate and reproducible results.(1,2) With the positive cell detection command, it is possible to identify the positive (and negative cells) in a digital slide and subsequently calculate the percentage of positive cells.

The first step of the positive cell detection is selecting the image type, which is based on the type of staining used. The second step is stain separation, for which Qupath uses the color deconvolution method, which separates the different stains and background of an image. The positive cell detection may become more accurate when the stains are adequately separated. Stain vectors are key in this method, as they are supposed to give a normalized representation of the color of each of the stains in the images. Stain vectors were set using a representative image, which is shown below.


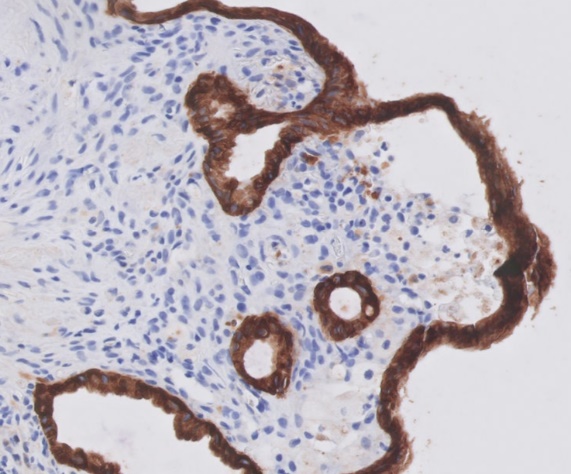


We have used the default settings of the positive cell detection script, except for the threshold variable which was set to 0.5 instead of 1.0, as this gave more accurate results i.e., better detection of both positive and negative cells.

The following script was run for all samples:

1. *setImageType('BRIGHTFIELD_H_DAB');*
2. *setColorDeconvolutionStains('{"Name" : "H-DAB modified 2", "Stain 1" : "Hematoxylin", "Values 1" : "0.75035 0.59012 0.29789", "Stain 2" : "DAB", "Values 2" : "0.28639 0.57929 0.76316", "Background" : " 225 224 229"}');*
3. *selectAnnotations();*
4. *runPlugin('qupath.imagej.detect.cells.PositiveCellDetection', '{"detectionImageBrightfield":"Hematoxylin OD","requestedPixelSizeMicrons":0.5,"backgroundRadiusMicrons":8.0,"backgroundByReconstruction":true,"medianRadiusMicrons":0.0,"sigmaMicrons":1.5,"minAreaMicrons":10.0,"maxAreaMicrons":400.0,"threshold":0.05,"maxBackground":2.0,"watershedPostProcess":true,"excludeDAB":false,"cellExpansionMicrons":5.0,"includeNuclei":true,"smoothBoundaries":true,"makeMeasurements":true,"thresholdCompartment":"Cell: DAB OD* mean","*thresholdPositive1":0.2,"thresholdPositive2":0.4,"thresholdPositive3":0.6000000000000001,"singleThreshold":true}')*

**References:**

1. Bankhead P, Loughrey MB, Fernández JA, Dombrowski Y, McArt DG, Dunne PD, et al. QuPath: Open source software for digital pathology image analysis. Sci Rep [Internet]. 2017;7(1):16878. Available from: https://doi.org/10.1038/s41598-017-17204-5

2. https://qupath.readthedocs.io/en/0.4/index.html [Internet]. 2023 [cited 2023 Apr 22]. Available from: https://qupath.readthedocs.io/en/0.4/index.html
